# Postoperative pain after single-visit root canal treatments in necrotic teeth comparing instruments’ kinematics and apical instrumentation limits – a prospective randomized multicenter clinical trial

**DOI:** 10.1186/s12903-024-04225-6

**Published:** 2024-04-20

**Authors:** Ricardo Machado, Guilherme Moreira, Daniel Comparin, Arthur Pimentel Barroso, Jaqueline Nascimento, Caio Cézar Randi Ferraz, Sérgio Aparecido Ignácio, Lucas da Fonseca Roberti Garcia, Rodrigo Rodrigues Amaral, David Shadid, Ulisses Xavier da Silva Neto

**Affiliations:** 1 College of Dentistry, Department of Restorative Sciences, Division of Endodontics, Health Sciences Center, University of Oklahoma - COD/OUHSC, Oklahoma City, Oklahoma USA; 2 Clinical Practice Limited to Endodontics, Francisco Beltrão, Paraná Brazil; 3 Clinical Practice Limited to Endodontics, Cunha Porã and Francisco Beltrão, Paraná, Brazil; 4 Piracicaba Dental School, Department of Restorative Dentistry, Division of Endodontics, State University of Campinas – FOP/UNICAMP, Piracicaba, São Paulo Brazil; 5 School of Dentistry, Department of Endodontics, Pontifical Catholic University of Paraná – PUC/PR, Curitiba, Paraná Brazil; 6 School of Dentistry, Department of Statistics, Pontifical Catholic University of Paraná – PUC/PR, Curitiba, Paraná Brazil; 7 Department of Dentistry, Division of Endodontics, Federal University of Santa Catarina – UFSC, Florianópolis, Santa Catarina Brazil; 8 College of Medicine and Dentistry, James Cook University – JCU, Smithfield, Cairns, Australia

**Keywords:** Apical instrumentation limit, Asymptomatic apical periodontitis, Root canal treatment, Instruments' kinematics, Intentional foraminal enlargement, Postoperative pain

## Abstract

**Objectives:**

This prospective randomized multicenter clinical trial (PRMCT) investigated postoperative pain after single-visit root canal treatments in teeth affected by pulp necrosis (PN), and asymptomatic apical periodontitis (AAP) (with apical radiolucent areas) or normal periradicular tissues (without apical radiolucent areas) comparing different instruments' kinematics and apical instrumentation limits.

**Methods:**

Before chemomechanical preparation, 240 patients/teeth were randomly distributed into four groups (*n* = 60) according to the instruments' kinematics (rotary or reciprocating) and apical instrumentation limits (with or without intentional foraminal enlargement [IFE]). After that, specimens were submitted to the same irrigation and obturation techniques, and the patients were referred to undergo the definitive restorations. No medication was prescribed, but the patients were instructed to take either paracetamol (750 mg every 6 h for three days) or ibuprofen (600 mg every 6 h for three days) in pain cases. Postoperative pain incidence and levels were assessed at 24-, 48-, and 72 h following treatment completion according to a verbal rating scale (VRS) following a score. The Kolmogorov–Smirnov test was applied to assess the normality of the data. Mann–Whitney U, Chi-square, Friedman's ANOVA, and Friedman's multiple 2 to 2 comparison tests were employed to identify potential significant statistical differences among the variables in the study groups (*P* < .05).

**Results:**

Significant statistical differences were only observed among the groups considering tooth, periradicular status, and the occurrence of overfilling (sealer extrusion) (*P* < 0.00). Patients with teeth instrumented through rotary kinematics and without IFE experienced lower rates of postoperative pain; however, this difference was relevant only at 24 h (*P* < 0.05).

**Conclusions:**

Postoperative pain was lower after using a rotary file system (Profile 04) inserted up to the apical constriction (AC). However, this finding was just statistically meaningful at 24 h.

**Trial registration:**

This PRMCT was approved by the Human Research Ethics Committee of the Paranaense University – UNIPAR, Francisco Beltrão, PR, Brazil (CAAE. 46,774,621.6.0000.0109) on 02/09/2021. It was registered at The Brazilian Registry of Clinical Trials – ReBEC (RBR-3r967t) on 01/06/2023, was performed according to the Principles of the Helsinki Declaration and is reported following the Consolidated Standards of Reporting Trials Statement.

## Background

Root canal treatment is based on cleaning, shaping, and filling the root canal system (RCS) to maintain or restore the health of periapical tissues [1]. Chemomechanical preparation [2], and intracanal dressing [3] (when used) are the main responsible for the disinfection process; nevertheless, the complete eradication of endodontic infection is unfeasible due to the following primary and synergic factors: i) the anatomical complexity of the RCS [4], and; ii) the virulence and resistance of endodontic pathogens, mainly when they are organized in biofilms [5].

Efficient biomechanical preparation is only achieved by determining a correct apical limit, defined as the distance between two opposite external and internal points/surfaces. While the external point is located on the coronary surface, the internal point corresponds to the greatest depth reached by the endodontic files used during the root canal shaping [6, 7].

Among the main factors associated with the endodontic prognosis, determining apical instrumentation limits close to the cement-dentin-canal junction plays a role in obtaining favorable outcomes [7–12]. Therefore, despite different philosophical trends [13, 14], scientific evidence recommends the working length be set at 0.5–1.0 mm from the major apical foramina, i.e., at the apical constriction (AC). This recommendation is based on sound wound healing principles – the severance of the tissue in that area will create the smallest possible wound – the less tissue to heal, the better the cure [7].

However, microbiological analyses performed through molecular methods have revealed the existence of bacterial biofilms in the apical foramen (AF) [5], which represents the main reason for investigating the effects of intentional foraminal enlargement (IFE) on the prognosis of endodontic therapy [15].

IFE consists of widening the AF using an endodontic file larger than the anatomic constriction at the foramen level or beyond [14, 16, 17]. IFE aims to reduce bacterial content by eliminating contaminated cementum and dentin through the mechanical widening of the AF [14]. This approach has been investigated in past studies, which signaled the possibility of a greater bacterial reduction in the foraminal region, potentially associated with improved endodontic prognosis. The available clinical evidence reports a success rate of up to 96% using this approach [18, 19]. Nonetheless, IFE may result in a more significant amount of debris being extruded [20] – an undesirable event potentially associated with postoperative pain from the induction of a local inflammatory process influenced by several factors, such as irrigation solutions or techniques [21], instrument's kinematics [22], as well as apical instrumentation limits [23].

To date, no study has been performed to investigate the incidence and levels of postoperative pain after single-visit root canal treatments in teeth affected by pulp necrosis (PN), and asymptomatic apical periodontitis (AAP) (with apical radiolucent areas) or normal periradicular tissues (without apical radiolucent areas) comparing different instruments' kinematics (rotary or reciprocating) and apical instrumentation limits (with or without IFE). Accordingly, this prospective randomized multicenter clinical trial (PRMCT) was planned to investigate these factors. The null hypothesis established was that the instruments' kinematics and apical instrumentation limits would not affect the level and frequency of postoperative pain after single-visit root canal treatments in teeth with the clinical features described above.

## Methods

This PRMCT was approved by the Human Research Ethics Committee of the Paranaense University – UNIPAR, Francisco Beltrão, PR, Brazil (CAAE. 46,774,621.6.0000.0109) on 02/09/2021. It was registered at The Brazilian Registry of Clinical Trials – ReBEC (RBR-3r967t) on 01/06/2023, was performed according to the Principles of the Helsinki Declaration [24], and is reported following the Consolidated Standards of Reporting Trials Statement [25]. The patients received information about postoperative care, clinical and radiographic exams, and alternative treatment options. All of them (or caregivers for those under 18) were given details about the study and treatment protocol, and informed consent was obtained. Consent for publication was not applicable to this research.

### Sample size calculation

The sample size for this research was determined after a pilot study, in which less than 5% of patients reported significant postoperative pain (acute, severe, or moderate) after the treatment. Considering a confidence level of 95% and a maximum margin of error of 5.5%, the proportion-sampling method determined a sample size of 240 patients/teeth (60 per group) [26].

### Case selection

This PRMCT was conducted on individuals aged 14 to 86 (mean ± SD = 40.39 ± 6.35) between July 2022 and July 2023. The adopted inclusion criteria were teeth affected by pulp necrosis, and AAP (with apical radiolucent areas) or normal periradicular tissues (without apical radiolucent areas), physiological periodontal probing depth (≤ 3 mm), previously submitted to the endodontic access, and subsequently referred for root canal treatment. As all teeth had been previously open, the diagnosis of PN was based on the following criteria/information/signs: i) all the referral letters provided by the indicators showed this diagnosis (PN); ii) some teeth presented chronic apical periodontitis visible radiographically; iii) all teeth presented negative responses to the cold (EndoIce, Coltene/Whaledent Inc., Cuyahoga Falls, Ohio, United States) and electric pulp tests (Diagnostic Unit, Sybron Endo, Orange, United States of America), and; iv) all treated teeth present complete absence of bleeding during the treatment. Exclusion criteria concerning personal, behavioral, emotional, and systemic conditions of the patients were: recent use of anti-inflammatories, analgesics, or antibiotics; presence of trismus and systemic diseases; intolerance to the use of non-steroidal anti-inflammatory drugs; lack of cooperation, and pregnancy. Exclusion criteria considering odontogenic factors were: teeth affected by root resorptions, associated with sinus tracts, presenting periodontal compromise (probing depth > 3 mm), previously traumatized, incorrectly positioned (malocclusion), and under occlusal trauma [26]. Each patient had only one tooth included in the study. Four experienced endodontists performed the treatments in specialized clinics following a previously written descriptive protocol for each study group [27].

### Randomization, allocation concealment of the instrumentation systems, and pretreatment instructions

**Fig. 1:**
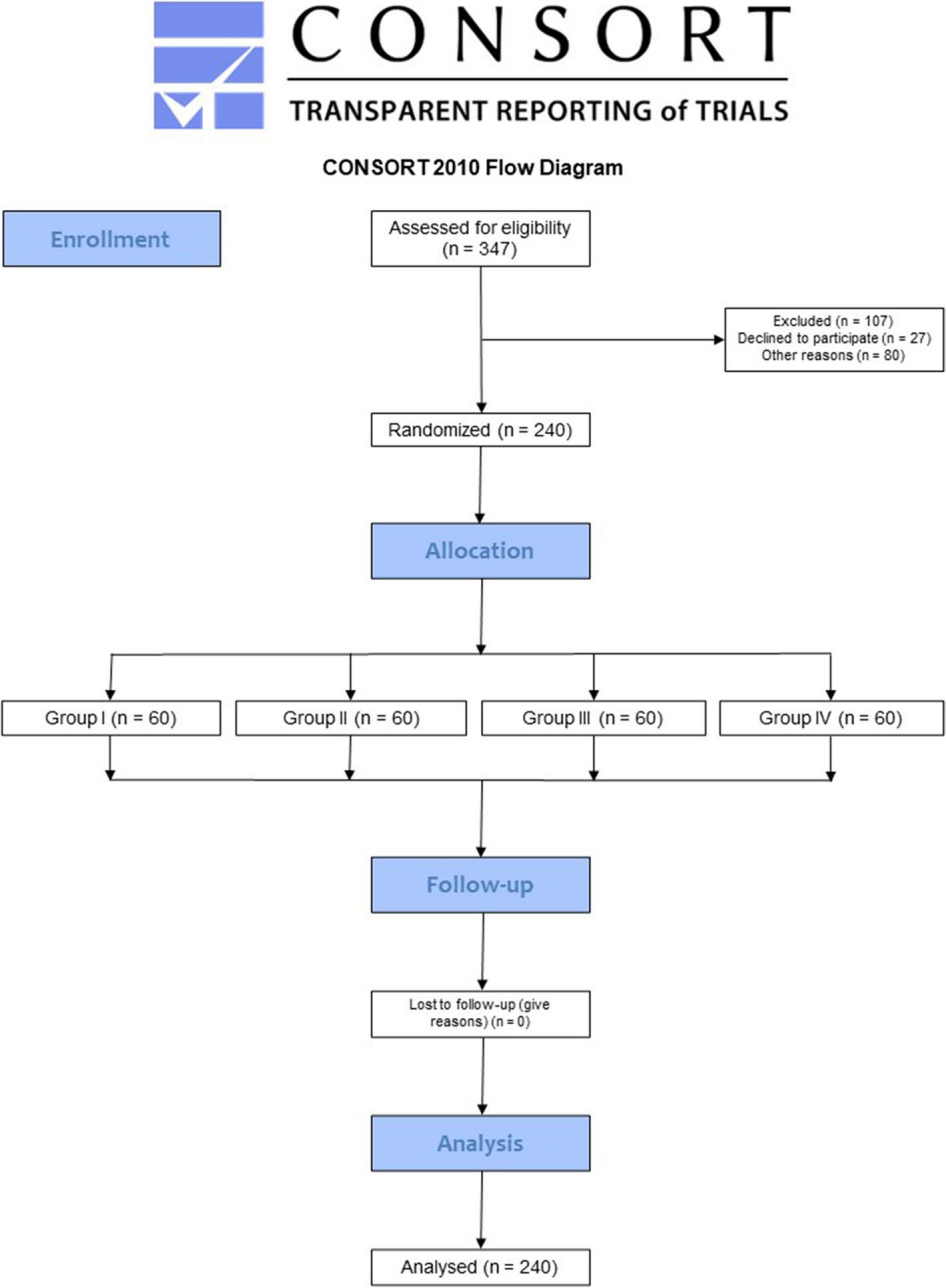
Study flowchart

The randomization process was done using a table created by the Sealed Envelope™ software (www.sealedenvelope.com – Exmouth House, London, UK). The task was carried out by an investigator not involved in the present research. A list of 240 numbers was prepared and distributed into three blocks (80 per group). First, each number corresponding to a study group was placed in a numbered, opaque, and sealed envelope. When a patient was deemed eligible, the envelope was opened before the root canal treatment to determine the necessary clinical procedures. This way, the four clinicians performed 15 root canal treatments that composed the specimens of each study group (n. 60), totaling 240 patients/teeth (total sample). Based on the previously stated inclusion and exclusion criteria, Fig. 1 exposes the study flow chart.

### Treatment protocol

Following clinical and radiographic examinations of each patient, the tooth was anesthetized using 2% mepivacaine with epinephrine 1:100.000 (Mepiadre; DFL Indústria e Comércio S.A., Rio de Janeiro, RJ, Brazil). After placing and disinfecting the rubber dam, the temporary restoration was removed using nos. 1014 or 1016 HL burs (KG Sorensen, Barueri, SP, Brazil). After reaching the pulp chamber, 5 mL of 2.5% sodium hypochlorite (NaOCl) (Fórmula & Ação, São Paulo, SP, Brazil) was used for irrigation by using a NaviTip 31 G needle (Ultradent, South Jordan, UT, United States of America). Initial exploration of the root canal was performed with no. 10 or 15 K-FlexoFiles (Dentsply-Maillefer, Ballaigues, Switzerland). The cervical and middle thirds were prepared with Gates-Glidden drills (Dentsply-Maillefer) activated by an endodontic electric motor (X-Smart Plus, Dentsply-Maillefer) at 800 rpm. Before the chemomechanical preparation, anatomical diameters of the AF and AC were identified through K-FlexoFiles in ascending order to plan and establish similar apical preparation sizes regardless of the study groups (Tables 1 and 2).

**Table 1:** Study groups

Group	Protocols
I	Chemomechanical preparation with a rotary motion by using Profile 04 system files (Dentsply-Maillefer) driven by the X-Smart Plus electric motor (Dentsply-Maillefer) at 300 rpm with a torque setting of 2 N.cm, with a slow in-and-out pecking motion that did not exceed 3–4 mm in amplitude, according to the crown-down philosophy without IFE, i.e., the apical limit was established around the AC (-0.5 mm from the AF identified by the apex locator^a^ as 0.0 mm). A 2.5 mL aliquot of 2.5% NaOCl was used as an irrigating solution at each file use or change, applied with a NaviTip 31 G needle (Ultradent) up to 5 mm short of the AF. The same amount of NaOCl was used for each canal (40 mL)
II	The exact specifications described for Group I, but with IFE, i.e., the apical limit was established at 0.5 mm beyond the AF (identified by the apex locator^a^ as 0.0 mm)
III	Chemomechanical preparation with a reciprocating motion by using Reciproc system files (VDW) driven by the X-Smart Plus electric motor (Dentsply-Maillefer), with a slow in-and-out pecking motion that did not exceed 3–4 mm in amplitude without IFE, i.e., the apical limit was established around the AC (-0.5 mm from the AF identified by the apex locator^a^ as 0.0 mm). A 2.5 mL aliquot of 2.5% NaOCl was used as an irrigating solution at each file use or change, applied with a NaviTip 31 G needle (Ultradent) up to 5 mm short of the AF. The same amount of NaOCl was used for each canal (40 mL)
IV	The exact specifications described for Group III, but with IFE, i.e., the apical limit was established at 0.5 mm beyond the AF (identified by the apex locator^a^ as 0.0 mm)

^*a*^*Root ZX (J Morita, Tokyo, Japan)*

**Table 2:** The chemomechanical preparation planning for the study groups

	**Group I**	**Group II**		**Group III**	**Group IV**	
**Approximate size of the AC/AF before the chemomechanical preparation (corresponding to the manual FlexoFile number)**	**The final file used around the AC** **(-0.5 mm from the AF) (tip/taper)**	**The final file used at 0.5 mm beyond** **the AF (tip/taper)**	**Approximate size of AC after the chemomechanical preparation**	**The final file used around the AC** **(-0.5 mm from the AF) (tip/taper)**	**The final file used at 0.5 mm beyond** **the AF (tip/taper)**	**Approximate size of AC after the chemomechanical preparation**
10/12	25/04	25/04	29	25/08	25/08	33
15/17	40/04	40/04	44	40/06	40/06	46
20/22	40/04	40/04	44	40/06	40/06	46
25/27	50/04	50/04	54	50/05	50/05	55
30/32	50/04	50/04	54	50/05	50/05	55

After chemomechanical preparation, the root canals were irrigated with 3 mL of 17% EDTA (Fórmula & Ação) for 3 min, followed by a final rinse with 5 mL of saline solution by using a NaviTip 31 G needle (Ultradent), inserted up to 5 mm from the AF, and dried with absorbent paper points (Dentsply-Maillefer).

For the root canal filling, the main gutta-percha cone corresponding to the master apical file was calibrated and stabilized at the AC and 1.5 mm from the AF for G1 and G3 and G2 and G4, respectively. This strategy was based on the greater possibility of gutta-percha extravasation to the periradicular tissues, considering the IFE had been carried out in teeth from G2 and G4.

After confirming the radiographic obturation limit, the main gutta-percha cone was coated with a zinc oxide-based sealer (Endofill, Dentsply Indústria e Comércio Ltda., Pirassununga, SP, Brazil), inserted into the root canal, and submitted to the thermocompaction process (Tagger's hybrid technique). After cleaning the pulp chamber, the following steps were conducted: i) provisional restoration of endodontic access with a temporary restorative material (Cavitec, Caitech, São José dos Pinhais, PR, Brazil); ii) occlusal adjustment (wholly taken out of occlusion); iii) final periapical radiography, and iv) referral of patients to perform definitive restoration. No medication was prescribed; however, the patients were instructed to take either 750 mg of paracetamol or 600 mg of ibuprofen every 6 h for three days in pain cases [28].

### Analysis of postoperative pain

A dental assistant not involved in the treatment procedures contacted each patient by phone 24-, 48-, and 72 h post-treatment to assess their pain, which was classified according to a verbal rating scale (VRS) following a score (Table 3) [29]. The collected information was entered into a spreadsheet.

**Table 3:** The scoring system based on a verbal rating scale used in the study

Score	Pain	Features
0	No pain	No discomfort or pain
1	Slight pain	The patient could be distracted from feeling pain, and no analgesia was required
2	Moderate pain	The patient felt moderate pain even while concentrating on some other activity, and an analgesic was required
3	Severe pain	The patient could no longer perform any activity and needed to lie down and rest (analgesics had little or no effect on pain relief)

### Statistical analysis

The Statistical Package for the Social Sciences Version 25.0 (SPSS Inc, Chicago, IL, United States of America) was used for the statistical analysis. The Kolmogorov–Smirnov test was applied to assess the normality of the data. Mann–Whitney U, Chi-square, Friedman's ANOVA, and Friedman's multiple 2 to 2 comparison tests were employed to identify potential significant statistical differences among the variables in the study groups (*P* < 0.05) [30].

## Results

Clinical and demographic data from the patients/teeth that constituted the sample of the current PRMCT and their respective statistical analyses are exposed in Table 4. Significative statistical differences among the groups were only observed considering tooth, periradicular status, and the incidence of overfilling (sealer extrusion) (*P* < 0.00). Of 60 teeth of G3, 27 (45%) were first mandibular molars; meanwhile, in G1, G2, and G4, only 7 (11.7%), 8 (13.3%), and 5 (8.3%) first mandibular molars were present, respectively. Concerning periradicular status, in G1, 44 (73.3%) teeth showed AAP (with apical radiolucent areas). In G2, G3, and G4, 12 (20%), 40 (66.7%), and 27 (45%) teeth presented the same diagnosis in that order. Sealer extrusion (overfilling) happened in only 3 (5%) teeth from G1. In G2, G3, and G4, this event did occur in 24 (40%), 14 (23.3%), and 26 (43.3%) teeth, respectively.

**Table 4:** Demographic and clinical data evaluated in the study considering the groups

	**Groups**					
**Variable**	**I (n. 60)**	**II (n. 60)**	**III (n. 60)**	**IV (n. 60)**	**Total (n. 240)**	***P* value**
**Age (mean ± standard deviation)**	40.6 ± 18.29^a^	38.48 ± 16.49^a^	41.37 ± 15.30^a^	41.10 ± 15.4^a^	40.39 ± 16.35^a^	0.76
< 30 years-old (n. / % considering the age / % considering the group)	22 / 27.8% / 36.7%^a^	20 / 25.3% / 33.3%^a^	16 / 20.3% / 26.7%^a^	21 / 26.6% / 35%^a^	79 / 100% / 32.9%^a^	
≥ 30 and ≤ 50 years-old (n. / % considering the age / % considering the group)	22 / 25% / 36.7%^a^	22 / 25% / 36.7%^a^	28 / 31.8% / 46.7%^a^	16 / 18.2% / 26.7%^a^	88 / 100% / 36.7%^a^	
≥ 50 years-old (n. / % considering the age / % considering the group)	16 / 21.9% / 26.7%^a^	18 / 24.7% / 30%^a^	16 / 21.9% / 26.7%^a^	23 / 31.5% / 38.3%^a^	73 / 100% / 30.4%^a^	
Total	60 / 25% / 100%	60 / 25% / 100%	60 / 25% / 100%	60 / 25% / 100%	240 / 100% / 100%	
**Gender**						
Male (n. / % considering the gender / % considering the group)	27 / 26.7% / 45%^a^	28 / 27.7% / 46.7%^a^	22 / 21.8% / 36.7%^a^	24 / 23.8% / 40%^a^	101 / 100% / 42.1%^a^	0.66
Female (n. / % considering the gender / % considering the group)	33 / 23.7% / 55%^a^	32 / 23% / 53.3%^a^	38 / 27.3% / 63.3%^a^	36 / 25.9% / 60%^a^	139 / 100% / 57.9%^a^	
Total	60 / 25% / 100%	60 / 25% / 100%^a^	60 / 25% / 100%	60 / 25% / 100%	240 / 100% / 100%	
**Teeth**						
Lower canine (n. / % considering the tooth / % considering the group)	1 / 14.3% / 1.7%ª	0 / 0% / 0%ª	2 / 28.6% / 3.3%ª	4 / 57.1% / 6.7%ª	7 / 100% / 2.9%	** < 0.00**
Upper canine (n. / % considering the tooth / % considering the group)	5 / 45.5% / 8.3%ª	3 / 27.3% / 5%ª	2 / 18.2% / 3.3%ª	1 / 9.1% / 1.7%ª	11 / 100% / 4.6%	
Lower central incisor (n. / % considering the tooth / % considering the group)	2 / 25% / 3.3%ª	4 / 50% / 6.7%ª	0 / 0% / 0%ª	2 / 25% / 3.3%ª	8 / 100% / 3.3%	
Upper central incisor (n. / % considering the tooth / % considering the group)	6 / 35.3% / 10%ª	7 / 41.2% / 11.7%ª	1 / 5.9% / 1.7%ª	3 / 17.6% / 5%ª	17 / 100% / 7.1%	
Lower lateral incisor (n. / % considering the tooth / % considering the group)	1 / 12.5% / 1.7%ª	1 / 12.5% / 1.7%ª	4 / 50% / 6.7%ª	2 / 25% / 3.3%ª	8 / 100% / 3.3%	
Upper lateral incisor (n. / % considering the tooth / % considering the group)	6 / 66.7% / 10%ª	1 / 11.1% / 1.7%ª	1 / 11.1% / 1.7%ª	1 / 11.1% / 1.7%ª	9 / 100% / 3.8%	
**Mandibular first molar (n. / % considering the tooth / % considering the group)**	7 / 14.9% / 11.7%ª	8 / 17% / 13.3%ª	**27 / 57.4% / 45%**^**b**^	5 / 10.6% / 8.3%ª	47 / 100% / 19.6%	
Maxillary first molar (n. / % considering the tooth / % considering the group)	11 / 40.7% / 18.3%ª	5 / 18.5% / 8.3%ª	5 / 18.5% / 8.3%ª	6 / 22.2% / 10%ª	27 / 100% / 11.3%	
Mandibular first premolar (n. / % considering the tooth / % considering the group)	2 / 12.5% / 3.3%ª	2 / 12.5% / 3.3%ª	2 / 12.5% / 3.3%ª	10 / 62.5% / 16.7%ª	16 / 100% / 6.7%	
Maxillary first premolar (n. / % considering the tooth / % considering the group)	1 / 5.9% / 1.7%ª	6 / 35.3% / 10.0%ª	7 / 41.2% / 11.7%ª	3 / 17.6% / 5.0%ª	17 / 100% / 7.1%	
Lower second molar (n. / % considering the tooth / % considering the group)	6 / 42.9% / 10%ª	1 / 7.1% / 1.7%ª	4 / 28.6% / 6.7%ª	3 / 21.4% / 5%ª	14 / 100% / 5.8%	
Maxillary second molar (n. / % considering the tooth / % considering the group)	4 / 30.8% / 6.7%ª	4 / 30.8% / 6.7%ª	1 / 7.7% / 1.7%ª	4 / 30.8% / 6.7%ª	13 / 100% / 5.4%	
**Lower second premolar (n. / % considering the tooth / % considering the group)**	5 / 19.2% / 8.3%^a.b^	7 / 26.9% / 11.7%^a.b^	**1 / 3.8% / 1.7%**^**b**^	13 / 50% / 21.7%^a^	26 / 100% / 10.8%	
Maxillary second premolar (n. / % considering the tooth / % considering the group)	3 / 15% / 5%^a^	11 / 55% / 18.3%^a^	3 / 15% / 5%^a^	3 / 15% / 5%^a^	20 / 100% / 8.3%	
Total	60 / 25% / 100%	60 / 25% / 100%	60 / 25% / 100%	60 / 25% / 100%	240 / 100% / 100%	
**Periradicular status**						
AAP with apical radiolucent areas (n. / % of this periradicular status considering each tooth / % of this periradicular status considering the group	**44 / 35.8% / 73.3%ª**	12 / 9.8% / 20%^b^	40 / 32.5% / 66.7%^a.c^	27 / 22% / 45%^c^	123 / 100% / 51.3%	** < 0.00**
AAP with normal periradicular tissues (without apical radiolucent areas) (n. / % of this periradicular status considering each tooth / % of this periradicular status considering the group	**16 / 13.7% / 26.7%ª**	48 / 41% / 80%^b^	20 / 17.1% / 33.3%^a.c^	33 / 28.2% / 55.0%^c^	117 / 100% / 48.8%	
Total	60 / 25% / 100%	60 / 25% / 100%	60 / 25% / 100%	60 / 25% / 100%	240 / 100% / 100%	
**Overfilling/sealer extrusion**						
Yes (n. / % of overfilling/sealer extrusion considering all the teeth / % of overfilling/sealer extrusion considering the group)	**3 / 4.5% / 5%**^**a**^	24 / 35.8% / 40%^b^	14 / 20.9% / 23.3%^b^	26 / 38.8% / 43.3%^b^	67 / 100% / 27.9%	** < 0.00**
No (n. / % of overfilling/sealer extrusion considering all the teeth / % of overfilling/sealer extrusion considering the group)	**57 / 32.9% / 95%ª**	36 / 20.8% / 60%^b^	46 / 26.6% / 76.7%^b^	34 / 19.7% / 56.7%^b^	173 / 100% / 72.1%	
Total	60 / 25% / 100%	60 / 25% / 100%	60 / 25% / 100%	60 / 25% / 100%	240 / 100% / 100%	

*- The p-value (P* < *0.05) provided by the Chi-Square Test indicates dependence between the two variables*

*- Different letters in the columns indicate a statistically significant difference between the columns for each category proved by the p-value* < *0.05) provided by the Z test of differences between two proportions with Bonferroni correction*

All patients (*n* = 240) could be evaluated during the three time frames (24-, 48-, and 72 h). Considering the incidence and degree of postoperative pain, G1 presented the lower levels; however, this difference was just observed at 24 h (*P* < 0.05). No significant differences were observed among the groups at 48 and 72 h (*P* ˃ 0.05) (Table 5).

**Table 5:** Pain levels considering the groups and time frames

		**Groups**					
**Time**	**Pain level**	**I**	**II**	**III**	**IV**	**Total**	***P* value**
**24 h**	None (n. / % according to the pain score / % regarding the group)	52 / 31%/ 86.7%^a^	43 / 25.6% / 71.7%^a.b^	38 / 22.6% / 63.3%^b^	35 / 20.8% / 58.3%^b^	168 / 100% / 70%	0.02
	Mild (n. / % according to pain score / % regarding group)	7/ 15.6% / 11.7%ª	8 / 17.8% / 13.3%ª	14 / 31.1% / 23.3%ª	16 / 35.6% / 26.7%ª	45 / 100% / 18.8%	
	Moderate (n. / % according to the pain score / % regarding the group)	1/ 5.3% / 1.7%ª	7 / 36.8% / 11.7%ª	5 / 26.3% / 8.3%ª	6 / 31.6% / 10%ª	19 / 100% / 7.9%	
	Acute/Severe (n. / % according to the pain score / % regarding the group)	0 / 0% / 0%ª	2 / 25% / 3.3%ª	3 / 37.5% / 5.0%ª	3 / 37.5% / 5%ª	8 / 100% / 3.3%	
	Total	60 / 25% / 100%	60 / 25% / 100%	60 / 25% / 100%	60 / 25% / 100%	240 / 100% / 100%	
**48 h**	None (n. / % according to the pain score / % regarding the group)	55 / 27.2% / 91.7%ª	46 / 22.8% / 76.7%ª	51 / 25.2% / 85%ª	50 / 24.8% / 83.3%ª	202 / 100% / 84.2%	0.49
	Mild (n. / % according to pain score / % regarding the group)	5 / 20% / 8.3%ª	9 / 36% / 15%ª	5 / 20% / 8.3%ª	6 / 24% / 10%ª	25 / 100% / 10.4%	
	Moderate (n. / % according to the pain score / % regarding the group)	0 / 0% / 0%ª	4 / 36.4% / 6.7%ª	4 / 36.4% / 6.7%ª	3 / 27.3% / 5%ª	11 / 100% / 4.6%	
	Acute/Severe (n. / % according to the pain score / % regarding the group)	0 / 0% / 0%ª	1 / 50% / 1.7%ª	0 / 0% / 0%ª	1 / 50% / 1.7%ª	2 / 100% / 0.8%	
	Total	60 / 25% / 100%	60 / 25% / 100%	60 / 25% / 100%	60 / 25% / 100%	240 / 100% / 100%	
**72 h**	None (n. / % according to the pain score / % regarding the group)	57 / 26.3% / 95%ª	53 / 24.4% / 88.3%ª	55 / 25.3% / 91.7%ª	52 / 24% / 86.7%ª	217 / 100% / 90.4%	0.27
	Mild (n. / % according to pain score / % regarding group)	3 / 20% / 5%ª	3 / 20% / 5%ª	2 / 13.3% / 3.3%ª	7 / 46.7% / 11.7%ª	15 / 100% / 6.3%	
	Moderate (n. / % according to the pain score / % regarding the group)	0 / 0% / 0%ª	3 / 42.9% / 5%ª	3 / 42.9% / 5%ª	1 / 14.3% / 1.7%ª	7 / 100% / 2.9%	
	Acute/Severe (n. / % according to the pain score / % regarding the group)	0 / 0% / 0%ª	1 / 100% / 1.7%ª	0 / 0% / 0.0%ª	0 / 0% / 0%ª	1 / 100% / 0.4%	
	Total	60 / 25% / 100%	60 / 25% / 100%	60 / 25% / 100%	60 / 25% / 100%	240 / 100% / 100%	

- The *p*-value (*P* < 0.05) provided by the Chi-Square Test indicates dependence between the two variables

- Different letters in the columns indicate a statistically significant difference between the columns for each category proved by the *p*-value < 0.05) provided by the Z test of differences between two proportions with Bonferroni correction

## Discussion

It has been suggested that the physical trauma caused by using fine instruments to unblock the AF during chemomechanical preparation (apical patency) would not be enough to drive or increase postoperative pain [31]. On the other hand, the procedure cannot effectively provide the disinfection of the AF or in its vicinity [15, 26], thus arousing a great interest of researchers about IFE. However, IFE may predispose to postoperative pain due to a virtually more significant apical extrusion of debris [20]. Since postoperative pain is a multifactorial event, this PRMCT was sought to investigate the levels and incidence of postoperative pain after single-visit root canal treatments performed in teeth affected by PN, and AAP (with apical radiolucent areas) or normal periradicular tissues (without apical radiolucent areas), comparing different instruments' kinematics (rotary or reciprocating) and apical instrumentation limits (with or without IFE). The null hypothesis was rejected because statistically significant differences were identified among the groups, whereas G1 presented lower pain levels 24 h after the treatments were concluded.

The study of the frequency and severity of postoperative pain after root canal treatment can be challenging due to the complexity of the matter [32, 33], so thorough methodological planning is crucial. After establishing the variables and hypothesis to be investigated and the number of treated patients needed to provide reliable results after performing the current PRMCT, it was concluded that it would be essential to identify the profile of patients of professionals responsible for carrying out the treatments. These professionals unanimously stated that most patients referred by their indicators presented teeth previously submitted to the endodontic access. Therefore, adopting this inclusion criterion would drastically optimize the time required to complete the investigation. Furthermore, including specimens previously submitted or not to endodontic access would represent a significant methodological bias since the coronary opening itself represents an important step towards reducing the bacterial load present in the RCS due to the removal of the pulp tissue from the pulp chamber, which is normally widely infected and, therefore, could strongly influence in the occurrence and intensity of postoperative pain. Accordingly, only patients with teeth previously submitted to endodontic access constituted the sample. With the same objective of controlling the occurrence of biases, only symptom-free patients were included to ensure accurate results, as preoperative pain has been found to predict postoperative pain [34]. Therefore, previous research has reported that multiple- and single-visit root canal treatments have shown similar incidences and levels of postoperative pain [34, 35] and healing of periapical tissues [36]. Nonetheless, in the present investigation, the treatments were carried out in a single session to reduce the number of clinical procedures and variables, such as intracanal dressing, which could compromise the analysis and reliability of the results [28, 37].

Various methods have been used to mensurate pain following root canal treatment, such as visual analog scales (VAS) [28, 38], (VRS) [39, 40], or both [41, 42]. Regardless of the method, it is essential to have an effective manner to ensure the patients can fully comprehend the questions and that the researchers can easily interpret the responses obtained [43]. A scoring system was used in this study to categorize the pain that patients experienced, based on a VRS, as follows: no pain, slight pain, moderate pain, and severe pain. The patients understood the categories, and this strategy is highly consolidated in the scientific literature [39, 40].

Overall, the postoperative pain scores were low, with only one patient from G2 (1.7%) reporting acute/severe pain 72 h after the treatment. Machado et al. [26] observed similar results using the same instrumentation system used herein for G2 (Profile 04) to conduct large intentional foraminal enlargement (LIFE) during chemomechanical preparation. The same root canal filling protocol was carried out compared to the current research, and only one patient (1.66%) reported acute/severe pain 72 h after the treatment. No patient has reported severe pain after 72 h in G3 and G4. According to Cruz Junior et al. [28], to ensure thorough disinfection of the apical third while minimizing the risk of debris being extruded with a reciprocating system (Reciproc), it is essential to use plenty of irrigation and perform frequent recapitulation of the root canal preparation. The same care was established during the treatments performed in this research to avoid an equivalent adverse event. This and the following clinical and therapeutic strategies likely were the main ones responsible for the general low occurrence and intensity of postoperative pain observed in this PRMCT: i) only teeth with PN were included in the sample; ii) all teeth were submitted to occlusal adjustment at the end of the root canal treatment, and; iii) regarding the irrigation protocol, the amount of irrigating solution used was considerable, and the tip of the irrigation needle was inserted into the canal only to a safe depth (-5 mm from the AF) to prevent the extravasation of the irrigation solutions to the periapical tissues [39], and; iv) experienced operators were responsible by conducting the treatments [28].

Moderate pain was reported by 8, 4, and 3 patients and by 11, 7, and 4 patients after 24-, 48-, and 72 h for rotary (G1 and G2) and reciprocating (G3 and G4) groups, respectively. Therefore, there was a trend for decreasing postoperative pain over time. However, paired analyses showed a statistically significant difference only between 24 and 72 h for the groups submitted to the rotary (G1 and G2) and reciprocating (G3 and G4) kinematics. These findings are consistent with those obtained by a prospective, randomized, double-blinded clinical trial performed by Shokraneh et al. [44] and a systematic review and meta-analysis conducted by Pak and White [45].

Nonetheless, Yaylali et al. [37] noted increased pain in teeth that underwent IFE 48 h after the treatment. This conflicting outcome could be due to the variations in the methodological designs between the studies. In the study by Yaylali et al. [37], root canal treatment was only performed on molars with PN and AAP. Chemomechanical preparation was conducted using the ProTaper Next system (Dentsply-Maillefer) after establishing the working length (WL) at the AF or 1 mm short from this measurement. In addition, the irrigation protocol consisted of a 2.5% NaOCl solution using a Max-I-Probe needle up to 2 mm from the WL, and a VAS was used to address the prevalence and levels of postoperative pain. The authors did not mention the estimated size of the AF. In the present PRMCT, anterior and posterior teeth diagnosed with PN, and AAP (with apical radiolucent areas) or normal periradicular tissues (without apical radiolucent areas) were treated; nonetheless, before the chemomechanical preparation, anatomical diameters of the AF and AC were identified with the aim of planning and establishing similar apical preparation sizes regardless of the study group. Therefore, the chemomechanical preparation was conducted with 2.5% NaOCl employing a NaviTip needle inserted up to 5 mm short of the AF. Afterward, postoperative pain was assessed using a VRS.

Based on the methodological design established to carry out this investigation, there were no significant statistical differences considering the instrumentation’s kinematics evaluated. These findings contrast with the study performed by Nekoofar et al. [46], which showed a lower difference in postoperative pain levels between patients treated with a rotary system (ProTaper Universal) and those treated with a reciprocating system (WaveOne). This disparity might be explained by relevant methodological differences observed in the study by Nekoofar et al. [46] and the current PRMCT, respectively, such as the diagnosis (irreversible pulpitis *versus* PN), irrigating solution (chlorhexidine *versus* NaOCl), systems used during chemomechanical preparation (ProTaper Universal/WaveOne *versus* Profile 04/Reciproc), apical instrumentation limits (0.5 mm short from the AF *versus* 0.5 mm short or beyond this point), the use of intracanal dressing (with *versus* without), the sealer and filling technique (AH 26/lateral compaction *versus* Endofill/Tagger's hybrid technique), and the methods used for the analysis of the postoperative pain (numerical rating scale *versus* VRS).

About the limitations of the present study, although PRMCTs are placed at the top of the “hierarchical scientific pyramid” used to classify different types of research according to the scientific power based on their methodological planning and design, some of the features of PRMCTs may lead to biased results. In the current study, significant statistical differences among the groups were only observed considering tooth, periradicular status, and the incidence of overfilling (sealer extrusion). From 60 teeth comprising each group's sample, in G3, 27 (45%) were mandibular first molars. In G1, G2, and G4, only 7 (11.7%), 8 (13.3%), and 5 (8.3%) mandibular first molars were present, respectively. Therefore, considering the anatomical complexity that may have influenced these results is a reasonable hypothesis. Concerning periradicular status, in G1, 44 (73.3%) teeth showed AAP (with apical radiolucent areas). In G2, G3, and G4, 12 (20%), 40 (66.7%), and 27 (45%) teeth presented the same diagnosis in that order. Since the presence of a periapical lesion represents the chronicity of an inflammatory process, the more significant number of teeth with AAP (with apical radiolucent areas) in G1 may have contributed to the lower incidence and levels of postoperative pain observed in this group. Sealer extrusion (overfilling) happened in only 3 (5%) teeth from G1. In G2, G3, and G4, this event did occur in 24 (40%), 14 (23.3%), and 26 (43.3%) teeth, respectively. Considering that higher rates of postoperative pain have already been associated with the use and extravasation of zinc oxide and eugenol-based sealers [47], the lower incidence of this event in the teeth of G1 may also have contributed to the lower incidence and levels of postoperative observed in this group.

Still about the limitations of the current scientific investigation, someone could say that it did not only compare two but three parameters capable of influencing the postoperative pain after endodontic treatments performed in teeth with PN, and AAP (with apical radiolucent areas) or normal periradicular tissues (without apical radiolucent areas) (kinematics, apical limit, and number of files used during the chemomechanical preparation). Thus, the latter factor could also have influenced the results presented herein. However, some reflections based on the results of well-planned previous research are essential. Silva et al. [48] investigated the amount of apically extruded debris produced by two full rotary systems (ProTaper Universal and ProTaper Next) compared to two single file reciprocating systems (WaveOne and Reciproc) after large apical preparations by using sixty mandibular premolars with a single canal, randomly assigned into four groups (n. 15). The ProTaper Universal system was associated with significantly more debris than the others (*P* < 0.05). No significant differences were found between ProTaper Next, WaveOne, and Reciproc systems (*P* > 0.05). De-Deus et al. [49] conducted a study to evaluate the amount of dentin debris quantitatively extruded from the apical foramen by comparing the full sequence of the ProTaper Universal system with the single-file ProTaper F2 used in reciprocating kinematics. Thirty mesial roots of lower molars were selected, and different instrumentation techniques resulted in 3 groups (n. 10 each). In G1, a crown-down hand-file technique was used, and in G2, a full sequence of the ProTaper Universal system was used. In G3, the ProTaper F2 file was used in a reciprocating motion. The apical preparation was equivalent to a 25 ISO size file. No significant difference was found in the amount of debris extruded between the full sequence of the ProTaper Universal system and the single-file ProTaper (F2) used in reciprocating kinematics (*P* > 0.05). In contrast, the hand instrumentation group extruded significantly more debris than both NiTi groups (*P* < 0.05). A prospective, parallel, randomized clinical trial conducted by Saber et al. [50], aimed to assess the effect of instrumentation kinematics (reciprocation [Wave One Gold] or continuous rotation [One Shape]) on bacterial reduction, postoperative pain, and incidence of flare-ups after root canal treatment of single-rooted mandibular premolars with AAP. Sixty-six patients were included in the study and were randomly allocated into two groups (n. 33) according to the studied systems. Under complete asepsis, bacterial samples were taken before (S1) and after (S2) a standard cleaning and shaping protocol. The bacterial reduction was evaluated using the culture technique and quantitative real-time polymerase chain reaction (qPCR) analysis. Postoperative pain was assessed using a VAS after 24-, 48-, and 72 h following treatment, while flare-ups were recorded and analyzed as a dichotomic variable (yes/no). The comparison between culture and qPCR methods showed that qPCR analysis demonstrated significantly higher pre-instrumentation baseline bacterial count (*P* < 0.05). The percentage of bacterial reduction, detected by either method, significantly decreased after instrumentation using both rotation and reciprocation kinematics (*P* < 0.05). However, the difference between the Wave One Gold and One Shape files was statistically non-significant (*P* > 0.05). The intra-group comparisons showed a significant reduction in postoperative pain with time (*P* < 0.05) for both groups. However, the inter-group comparison demonstrated that the difference in postoperative pain after the use of both systems was statistically non-significant (*P* > 0.05). The same occurred with the incidence of flare-ups (*P* = 1).

There is common sense that associates the extrusion of debris with postoperative pain in necrotic teeth, and IFE could contribute to that. However, Machado et al. [51] conducted a systematic review and meta-analysis to assess whether IFE was responsible for extruding more debris from extracted human teeth with fully formed apexes. Following the recommendations of Preferred Reporting Items for Systematic Review and Meta-Analysis – PRISMA, electronic and manual searches were performed to identify studies that evaluated the extrusion of debris, comparing different apical limits of instrumentation (with/without IFE). The quality of the studies selected was evaluated, and statistical analysis was conducted. Just three papers could be used to perform the meta-analysis. The heterogeneity was high, and the general risk of bias was moderate. However, there was no statistically significant difference in the extrusion of debris in teeth either submitted or not submitted to IFE.

Despite the *status quo* established around the subject, a careful and critical analysis is needed to analyze the association between the extrusion of debris and postoperative pain in Endodontics. Different instruments, kinematics, materials, substances, techniques, and apical limits should be studied. However, following Elmsallati et al. [52], besides the quantity of debris, the type and virulence of bacteria bound to debris and the resistance of host tissue are essential factors to be considered in this context. Therefore, the understanding that postoperative pain after endodontic procedures is a complex and extrinsic multifactorial phenomenon must be considered in future studies.

## Conclusion

According to the main findings of this PRMCT, postoperative pain was lower when the chemomechanical preparation was carried out using a rotary file system (Profile 04) inserted up to the AC. However, this finding was just statistically relevant at 24 h (*P* < 0.05). No significant differences were observed among the groups at 48 and 72 h.

## Data Availability

The datasets used and/or analyzed during the current study are available from the corresponding author upon reasonable request.

## References

[1] Gulabivala K, Ng YL (2023). Factors that affect the outcomes of root canal treatment and retreatment-A reframing of the principles. Int Endod J.

[2] Burklein S, Arias A: Effectiveness of root canal instrumentation for the treatment of apical periodontitis: A systematic review and meta-analysis. Int Endod J 2022. 10.1111/iej.13782 35670625

[3] Ordinola-Zapata R, Noblett WC, Perez-Ron A, Ye Z, Vera J (2022). Present status and future directions of intracanal medicaments. Int Endod J.

[4] Martins JNR, Marques D, Silva E, Carames J, Versiani MA (2019). Prevalence Studies on Root Canal Anatomy Using Cone-beam Computed Tomographic Imaging: A Systematic Review. J Endod.

[5] Siqueira JF, Rocas IN (2022). Present status and future directions: Microbiology of endodontic infections. Int Endod J.

[6] Ricucci D (1998). Apical limit of root canal instrumentation and obturation, part 1 Literature review. Int Endod J.

[7] Ricucci D, Langeland K (1998). Apical limit of root canal instrumentation and obturation, part 2 A histological study. Int Endod J.

[8] Dadresanfar B, Rotstein I (2021). Outcome of Endodontic Treatment: The Most Cited Publications. J Endod.

[9] Seltzer S, Bender IB, Turkenkopf S (1963). Factors Affecting Successful Repair after Root Canal Therapy. J Am Dent Assoc.

[10] Sjogren U, Figdor D, Persson S, Sundqvist G (1997). Influence of infection at the time of root filling on the outcome of endodontic treatment of teeth with apical periodontitis. Int Endod J.

[11] Sjogren U, Hagglund B, Sundqvist G, Wing K (1990). Factors affecting the long-term results of endodontic treatment. J Endod.

[12] Swartz DB, Skidmore AE, Griffin JA (1983). Twenty years of endodontic success and failure. J Endod.

[13] Borlina SC, de Souza V, Holland R, Murata SS, Gomes-Filho JE, Dezan Junior E, Marion JJ, Neto Ddos A (2010). Influence of apical foramen widening and sealer on the healing of chronic periapical lesions induced in dogs' teeth. Oral Surg Oral Med Oral Pathol Oral Radiol Endod.

[14] de Souza Filho FJ, Benatti O, de Almeida OP (1987). Influence of the enlargement of the apical foramen in periapical repair of contaminated teeth of dog. Oral Surg Oral Med Oral Pathol.

[15] Brandao PM, de Figueiredo JAP, Morgental RD, Scarparo RK, Hartmann RC, Waltrick SBG, Souza RA (2019). Influence of foraminal enlargement on the healing of periapical lesions in rat molars. Clin Oral Investig.

[16] Abada HM, Hashem AAR, Abu-Seida AM, Nagy MM (2022). The effect of changing apical foramen diameter on regenerative potential of mature teeth with necrotic pulp and apical periodontitis. Clin Oral Investig.

[17] Benatti O, Valdrighi L, Biral RR, Pupo J (1985). A histological study of the effect of diameter enlargement of the apical portion of the root canal. J Endod.

[18] Bourreau MLSF, M. R. S, Mota MJ. B. B., Zaia AA, de Lima CO, Prado M, Soares AJ. Evaluation of single visit endodontic treatment and non-surgical retreatment with foraminal enlargment of teeth with apical periodontitis. Rev Bras Odontol 2020, 77.

[19] Eyuboglu TF, Olcay K, Erkan E, Ozcan M (2020). Radiographic and Clinical Findings of Single-Visit Root Canal Treatments with Apical Enlargement in Necrotic Teeth: A Retrospective Cohort Study. Biomed Res Int.

[20] Tinaz AC, Alacam T, Uzun O, Maden M, Kayaoglu G (2005). The effect of disruption of apical constriction on periapical extrusion. J Endod.

[21] Adam M, Wootton J (2022). Conventional vs ultrasonic irrigation - which leads to less post-operative pain?. Evid Based Dent.

[22] Martins CM, De Souza Batista VE, Andolfatto Souza AC, Andrada AC, Mori GG, Gomes Filho JE (2019). Reciprocating kinematics leads to lower incidences of postoperative pain than rotary kinematics after endodontic treatment: A systematic review and meta-analysis of randomized controlled trial. J Conserv Dent.

[23] Borges Silva EA, Guimaraes LS, Kuchler EC, Antunes LAA, Antunes LS (2017). Evaluation of Effect of Foraminal Enlargement of Necrotic Teeth on Postoperative Symptoms: A Systematic Review and Meta-analysis. J Endod.

[24] General Assembly of the World Medical A: World Medical Association Declaration of Helsinki: ethical principles for medical research involving human subjects. J Am Coll Dent 2014, 81(3):14-18. 25951678

[25] Moher D, Hopewell S, Schulz KF, Montori V, Gotzsche PC, Devereaux PJ, Elbourne D, Egger M, Altman DG (2010). Consolidated Standards of Reporting Trials G: CONSORT 2010 Explanation and Elaboration: Updated guidelines for reporting parallel group randomised trials. J Clin Epidemiol.

[26] Machado R, Comparin D, Ignacio SA, da Silva Neto UX (2021). Postoperative pain after endodontic treatment of necrotic teeth with large intentional foraminal enlargement. Restor Dent Endod.

[27] Xavier F, Zuolo M, Nevares G, Kherlakian D, Velozo C, de Albuquerque D (2021). Postoperative Pain after Use of the WaveOne Gold and XP-endo Shaper Systems: A Randomized Clinical Trial. J Endod.

[28] Cruz Junior JA, Coelho MS, Kato AS, Vivacqua-Gomes N, Fontana CE, Rocha DG, da Silveira Bueno CE (2016). The Effect of Foraminal Enlargement of Necrotic Teeth with the Reciproc System on Postoperative Pain: A Prospective and Randomized Clinical Trial. J Endod.

[29] Pasqualini D, Mollo L, Scotti N, Cantatore G, Castellucci A, Migliaretti G, Berutti E (2012). Postoperative pain after manual and mechanical glide path: a randomized clinical trial. J Endod.

[30] Pagano M, Gauvreau K, Mattie H (2022). Principles of biostatistics.

[31] Abdulrab S, Rodrigues JC, Al-Maweri SA, Halboub E, Alqutaibi AY, Alhadainy H (2018). Effect of Apical Patency on Postoperative Pain: A Meta-analysis. J Endod.

[32] Sun C, Sun J, Tan M, Hu B, Gao X, Song J (2018). Pain after root canal treatment with different instruments: A systematic review and meta-analysis. Oral Dis.

[33] Nagendrababu V, Gutmann JL (2017). Factors associated with postobturation pain following single-visit nonsurgical root canal treatment: A systematic review. Quintessence Int.

[34] Riaz A, Maxood A, Abdullah S, Saba K, Din SU, Zahid S (2018). Comparison of frequency of post-obturation pain of single versus multiple visit root canal treatment of necrotic teeth with infected root canals. A Randomized Controlled Trial. J Pak Med Assoc.

[35] Wong AW, Zhang S, Li SK, Zhu X, Zhang C, Chu CH (2015). Incidence of post-obturation pain after single-visit versus multiple-visit non-surgical endodontic treatments. BMC Oral Health.

[36] Paredes-Vieyra J, Enriquez FJ (2012). Success rate of single- versus two-visit root canal treatment of teeth with apical periodontitis: a randomized controlled trial. J Endod.

[37] Yaylali IE, Teke A, Tunca YM (2017). The Effect of Foraminal Enlargement of Necrotic Teeth with a Continuous Rotary System on Postoperative Pain: A Randomized Controlled Trial. J Endod.

[38] Graunaite I, Skucaite N, Lodiene G, Agentiene I, Machiulskiene V (2018). Effect of Resin-based and Bioceramic Root Canal Sealers on Postoperative Pain: A Split-mouth Randomized Controlled Trial. J Endod.

[39] Relvas JB, Bastos MM, Marques AA, Garrido AD, Sponchiado EC (2016). Assessment of postoperative pain after reciprocating or rotary NiTi instrumentation of root canals: a randomized, controlled clinical trial. Clin Oral Investig.

[40] Cicek E, Kocak MM, Kocak S, Saglam BC, Turker SA (2017). Postoperative pain intensity after using different instrumentation techniques: a randomized clinical study. J Appl Oral Sci.

[41] Farzaneh S, Parirokh M, Nakhaee N, Abbott PV (2018). Effect of two different concentrations of sodium hypochlorite on postoperative pain following single-visit root canal treatment: a triple-blind randomized clinical trial. Int Endod J.

[42] Attar S, Bowles WR, Baisden MK, Hodges JS, McClanahan SB (2008). Evaluation of pretreatment analgesia and endodontic treatment for postoperative endodontic pain. J Endod.

[43] Arias A, Azabal M, Hidalgo JJ, de la Macorra JC (2009). Relationship between postendodontic pain, tooth diagnostic factors, and apical patency. J Endod.

[44] Shokraneh A, Ajami M, Farhadi N, Hosseini M, Rohani B (2017). Postoperative endodontic pain of three different instrumentation techniques in asymptomatic necrotic mandibular molars with periapical lesion: a prospective, randomized, double-blind clinical trial. Clin Oral Investig.

[45] Pak JG, White SN (2011). Pain prevalence and severity before, during, and after root canal treatment: a systematic review. J Endod.

[46] Nekoofar MH, Sheykhrezae MS, Meraji N, Jamee A, Shirvani A, Jamee J, Dummer PM (2015). Comparison of the effect of root canal preparation by using WaveOne and ProTaper on postoperative pain: a randomized clinical trial. J Endod.

[47] Khandelwal A, Jose J, Teja KV, Palanivelu A (2022). Comparative evaluation of postoperative pain and periapical healing after root canal treatment using three different base endodontic sealers - A randomized control clinical trial. J Clin Exp Dent.

[48] Silva EJ, Carapia MF, Lopes RM, Belladonna FG, Senna PM, Souza EM, De-Deus G (2016). Comparison of apically extruded debris after large apical preparations by full-sequence rotary and single-file reciprocating systems. Int Endod J.

[49] De-Deus G, Brandao MC, Barino B, Di Giorgi K, Fidel RA, Luna AS (2010). Assessment of apically extruded debris produced by the single-file ProTaper F2 technique under reciprocating movement. Oral Surg Oral Med Oral Pathol Oral Radiol Endod.

[50] Saber SM, Alfadag AMA, Nawar NN, Plotino G, Hassanien EE (2022). Instrumentation kinematics does not affect bacterial reduction, post-operative pain, and flare-ups: A randomized clinical trial. Int Endod J.

[51] Machado R, Vigarani G, Macoppi T, Pawar A, Glaci Reinke SM, Kovalik Goncalves AC (2021). Extrusion of debris with and without intentional foraminal enlargement - A systematic review and meta-analysis. Aust Endod J.

[52] Elmsallati EA, Wadachi R, Suda H (2009). Extrusion of debris after use of rotary nickel-titanium files with different pitch: a pilot study. Aust Endod J.

